# Cleavage by MMP‐13 renders VWF unable to bind to collagen but increases its platelet reactivity

**DOI:** 10.1111/jth.14729

**Published:** 2020-02-24

**Authors:** Joanna‐Marie Howes, Vera Knäuper, Jean‐Daniel Malcor, Richard W. Farndale

**Affiliations:** ^1^ Department of Biochemistry University of Cambridge Cambridge UK; ^2^ Cardiff University Dental School Cardiff UK

**Keywords:** glycoprotein Ib alpha, matrix metalloproteinase‐13, platelets, thrombosis, von Willebrand factor

## Abstract

**Background:**

Atherosclerotic plaque rupture and subsequent thrombosis underpin thrombotic syndromes. Under inflammatory conditions in the unstable plaque, perturbed endothelial cells secrete von Willebrand Factor (VWF) which, via its interaction with GpIbα, enables platelet rolling across and adherence to the damaged endothelium. Following plaque rupture, VWF and platelets are exposed to subendothelial collagen, which supports stable platelet adhesion, activation, and aggregation. Plaque‐derived matrix metalloproteinase (MMP)‐13 is also released into the surrounding lumen where it may interact with VWF, collagen, and platelets.

**Objectives:**

We sought to discover whether MMP‐13 can cleave VWF and whether this might regulate its interaction with both collagen and platelets.

**Methods:**

We have used platelet adhesion assays and whole blood flow experiments to assess the effects of VWF cleavage by MMP‐13 on platelet adhesion and thrombus formation.

**Results:**

Unlike the shear‐dependent cleavage of VWF by a disintegrin and metalloprotease with thrombospondin motif member 13 (ADAMTS13), MMP‐13 is able to cleave VWF under static conditions. Following cleavage by MMP‐13, immobilized VWF cannot bind to collagen but interacts more strongly with platelets, supporting slower platelet rolling in whole blood under shear. Compared with intact VWF, the interaction of cleaved VWF with platelets results in greater GpIbα upregulation and P‐selectin expression, and the thrombi formed on cleaved VWF–collagen co‐coatings are larger and more contractile than platelet aggregates on intact VWF‐collagen co‐coatings or on collagen alone.

**Conclusions:**

Our data suggest a VWF‐mediated role for MMP‐13 in the recruitment of platelets to the site of vascular injury and may provide new insights into the association of MMP‐13 in atherothrombotic and stroke pathologies.

**Essentials**

- Von Willebrand factor (VWF) exposure following plaque rupture tethers platelets to support adhesion, activation, and aggregation.
- Matrix metalloproteinase‐13 (MMP‐13) is also associated with and released upon plaque rupture.
- Unlike ADAMTS13, MMP‐13 is able to cleave VWF under static conditions.
- MMP‐13‐cleaved VWF binds more strongly to platelets forming denser thrombi on co‐coated collagen.

## 1 INTRODUCTION

Von Willebrand factor (VWF) is a large multimeric adhesive glycoprotein selectively produced in megakaryocytes (MKs) and endothelial cells (ECs).^1^ Patients with von Willebrand disease lack functional VWF protein and exhibit a moderate to severe hemorrhagic phenotype.^2^ Mature multimers of VWF are released into the blood from storage in Weibel‐Palade bodies in ECs and from α‐granules in activated platelets. The VWF protein has a multidomain structure comprising D1‐D2‐D′‐D3‐A1‐A2‐A3‐D4‐C1‐C2‐C3‐C4‐C5‐C6‐CK.^3^ Under static conditions, secreted VWF adopts a globular conformation, but under shear, unfolds to expose platelet and collagen binding sites^4^: The A1 domain is no longer protected by the D3 domain and can bind to GpIbα on the platelet surface, while both the A1 and A3 domains are able to bind to fibrillar collagens.^5^ VWF itself multimerizes to form highly thrombogenic ultra‐large multimers (UL‐VWF) which are in part regulated by a disintegrin and metalloprotease with thrombospondin motif (ADAMTS13), which binds to and under shear cleaves VWF at the A2 domain generating smaller, less reactive VWF aggregates.

Matrix metalloproteinases (MMPs) are proteolytic enzymes that mediate the degradation of many extracellular matrix and cell surface proteins, and are secreted as pro–enzymes that are activated following cleavage of the pro–peptide domain. Under inflammatory conditions such as those in the vulnerable plaque, increased MMP‐13 expression and release following plaque rupture^6^, ^7^, ^8^ brings the MMP into contact with plasma proteins, blood cells, and platelets. MMP‐13 is implicated in the early pathology of stroke progression, with plasma MMP‐13 levels reaching in excess of 10 ng/mL (200 nmol/L) in the blood of stroke patients.^9^, ^10^ A high plasma level of VWF is known to be associated with the development of cardiovascular disease and may predict stroke,^11^ while low levels of ADAMTS13 are associated with an increased risk of thrombosis and ischemic stroke.^12^ MMP levels have also been shown to be eight‐fold greater in atheromatous plaques than in normal vessels.^13^ Given that collagen and VWF are known to act synergistically in supporting platelet adhesion at the site of injury,^14^ it is not unreasonable to hypothesize that cleavage of VWF by MMP‐13 may serve to reduce the degree of platelet activation and adhesion in thrombus formation. In this study, we aimed to determine the effects of MMP‐13‐mediated degradation of VWF on platelet adhesion under both static and flow conditions. In contrast to the hypothesis above, we show here that while MMP‐13‐cleaved VWF can no longer bind to collagen, it provides a more adhesive and reactive substrate for platelets.

## 2 MATERIALS AND METHODS

### 2.1 MMP‐13 expression, purification, and activation

ProMMP‐13 was expressed, purified, activated, and dialyzed as previously described.^15^, ^16^, ^17^ ProMMP‐13 was activated using 1 mmol/L (final concentration) 4‐aminophenylmercuric acetate for 1 hour at 37°C prior to dialysis for 4 hours at 4°C. The structurally homologous but catalytically inactive MMP‐13(E204A) was a kind gift from Dr R. Visse (Kennedy Institute of Rheumatology Division, Imperial College London).^15^, ^16^ The (Cat)alytic domain of MMP‐13 (Δ249‐451) was expressed and purified from NS0 mouse myeloma cells as previously described.^18^ MMP‐13 GST‐Hemopexin (Hpx) domain was expressed in *E coli* using the pGEX‐2T expression vector, the forward primer TCCGCGTGGATCCCTCTATGGTCCAGGAGATGAA and the reverse primer GCAA‐ATTCCATTTTGTGGTGTTGAAGAATTCAT, which contain BamHI and EcoRI restriction sites, respectively, as previously described.^19^

### 2.2 Cleavage of VWF by MMP‐13

Purified human VWF (ab88533; abcam) at 0.2 mg/mL (final concentration in Tris pH 7.4) was incubated with MMP‐13 or ADAMTS13 (6156‐AD‐020; R&D Systems) at 1.5 µmol/L final enzyme concentration for 2 hours at 37°C. MMP‐13 alone was also incubated with Tris buffer at 37°C alongside the cleavage experiments in order to generate autolyzed (AL)MMP‐13 for use as a negative control. Reducing sample buffer was then added to the mixture prior to electrophoresis and Western blotting. Following incubation with MMP‐13, cleaved VWF was transferred onto polyvinylidene fluoride (PVDF) membrane, which was then stained with 0.1% Coomassie R250, 40% MeOH, 1% HAc to allow the visualization of protein bands and dried. The MMP‐13 cleavage sites on VWF were identified by Edman degradation using an ABI Procise 494HT Protein Sequencer^©^.

### 2.3 Electrophoresis and Western blotting

Protein samples in reducing sample buffer were boiled for 5 minutes and applied to 4%‐12% NuPage^®^ Gels and separated by electrophoresis using the Xcell SureLock™ system (Invitrogen) under reducing conditions. Proteins were then transferred on to nitrocellulose membrane (Millipore) at 80 V for 1 hour using a Hoefer semi‐dry blotting system. Following transfer, the membrane was blocked (5% BSA, 0.1% Tween 20 in TBS) for 1 hour. Rabbit anti‐VWF (ab9378; abcam) was incubated with the membrane overnight at 4°C at a dilution of 1:1000. Following washes with TBST, the membrane was incubated with horseradish peroxidase (HRP) conjugated goat anti‐rabbit at 1:10 000 dilution (P0448; Dako) for 1 hour at 24°C. The membrane was developed using a chemiluminescent substrate (RPN2209; GE Healthcare).

### 2.4 Collagen Toolkit peptide design and synthesis

Collagen Toolkit II and III peptides were generated using a CEM Liberty microwave‐assisted solid‐phase peptide synthesizer and N‐(9‐fluorenyl)methoxycarbonyl (Fmoc) chemistry as previously described.^19^, ^20^ All peptides were verified using matrix‐assisted laser desorption/ionization time‐of‐flight (MALDI‐TOF) mass spectrometry. Their triple‐helical conformation, verified by polarimetry, is maintained by the flanking sequences, GPC(GPP)_5_‐ and ‐(GPP)_5_‐GPC‐amide, at their N‐ and C‐terminus, respectively. For simplicity, peptides are referred to by their specific guest sequence. A negative control peptide, (GPC‐(GPP)_10_‐GPC‐amide), is referred to as GPP_10_.

### 2.5 Solid phase binding assays

#### 2.5.1 Collagen Toolkit assays

HB 96‐well plates (Nunc) were coated with either collagen Toolkit peptides or fibrillar or monomeric collagen at a saturating concentration (10 µg/mL in 0.01 mol/L acetic acid). All incubations were performed at 24°C for 1 hour unless otherwise stated in the presence of 2 mmol/L Mg^2+^. The wells were washed three times with adhesion buffer (1 mg/mL bovine serum albumin [BSA] in Tris‐buffered saline [TBS] containing 0.1% [v/v] Tween‐20) between each incubation step. Wells were blocked with 50 mg/mL BSA in TBS prior to the addition of VWF at a concentration of 5 µg/mL in adhesion buffer. Where indicated, intact VWF was pre‐incubated with 83 nmol/L MMP‐13(E204A) for 1 hour prior to adhesion assays.

Rabbit anti‐VWF raised against the whole molecule (abcam, Cambridge, UK), and goat anti‐rabbit HRP (Dako, Ely, UK) were added at a dilution of 1:2000 in adhesion buffer prior to the addition of tetramethylbenzadrine (TMB) substrate system (T0440; Sigma) and the plates read at 450 nm.

#### 2.5.2 Antibody affinity assay

HB 96‐well plates were coated with intact or cleaved VWF (5 µg/mL in TBS for 1 hour at 24°C. All further incubations were performed at room temperature for 1 hour unless otherwise stated. The wells were washed three times with adhesion buffer between each incubation step. The wells were then blocked with 50 mg/mL BSA in TBS prior to antibody addition and detection as described for collagen Toolkit assays.

#### 2.5.3 VWF recognition of MMP‐13

HB 96‐well plates were coated with 83 nmol/L proMMP‐13 in TBS for 1 hour at 24°C. All further incubations were performed as previously described for collagen Toolkit assays.

### 2.6 Washed platelet preparation and platelet adhesion xCELLigence assays

Platelets were purified and adhesion assays conducted as previously described.^21^, ^22^ Fibrous collagen type I was a gift from Ethicon Corp, Somerville, NJ, USA. Ninety‐six‐well xCELLigence E‐plates^®^ (ACEA Biosciences) were coated with type I collagen or intact or cleaved VWF (10 µg/mL) in phosphate‐buffered saline (PBS) for 1 hour at 24°C. Wells were also coated with autolyzed MMP (800 nmol/L) as a negative control. The wells were then blocked with 2 mg/mL BSA in TBS and 50 µL calcium‐free Tyrodes (CFT) buffer added as a baseline. Fifty microliter aliquots of 2 × 10^8^ platelets/mL in CFT were pre‐incubated for 1 hour with 1‐10 µmol/L of the αIIbβ3 inhibitor GR 144053 (4‐[4‐[4‐(aminoiminomethyl)phenyl]‐1‐piperazinyl]‐1‐piperidineacetic acid hydrochloride trihydrate; Calbiochem, Nottingham, UK) or 10‐50 µg/mL of the GpIbα inhibitor Myr‐Ser‐Ile‐Arg‐Tyr‐Ser‐Gly‐His‐Ser(PO_3_H_2_)‐Leu (ab143739; abcam) where indicated. xCELLigence experiments were allowed to proceed for 1 hour at 37°C and platelet binding measured as Cell Index.

### 2.7 Whole blood perfusion experiments

Blood from healthy medication‐free volunteers was collected into 40 μmol/L PPACK and supplemented hourly with 10 µmol/L PPACK. Blood was incubated with 1 μmol/L 3,3′dihexyloxacarbocyanine iodide (DiOC6) for 15 minutes before use. Blood was pre‐incubated with 10 µmol/L GR 144053 or 50 µg/mL Myr‐Ser‐Ile‐Arg‐Tyr‐Ser‐Gly‐His‐Ser(PO_3_H_2_)‐Leu for 1 hour where indicated. Blood was perfused over 10 µg/mL VWF and/or type I fibrous collagen‐coated slide as previously described.^21^, ^23^ Where indicated, slides were coated with (AL)MMP‐13 alone as a (negative) control. Thrombi were measured using an UplanFLN 40 × NA1.30 oil immersion objective and a field size of 360 × 360 μm on an Olympus FV300 confocal microscope.

Blood was perfused at a shear rate of 1000 seconds for 5 minutes with images being acquired every 5 seconds at the plane of the thrombogenic surface. Images were exported to ImageJ1.35 (National Institutes of Health) for analysis. Thresholding the coverslip plane optimized contrast; a manipulation which when applied to all images allowed measurements of particle size and count. Thrombus area covering the coverslip plane yielded the primary measurement of surface coverage (SC). Z‐stacks (sequential vertical images of a given field; ΔZ = 0.69 μm, and encompassing the entire thrombus height) were used to calculate thrombus volume as the sum of the detected surface areas of all images of the Z‐stack, multiplied by ΔZ. This value is divided by the field area (giving units of μm^3^/μm^2^) providing a free‐standing measure of thrombus formation, and although a volume measurement has the units of microns and is referred to as mean thrombus height. A separate measure of the absolute height of the thrombus, ZV50, was calculated as the Z‐height at which thrombus volume was half‐maximal. Platelet rolling measurements were taken as previously described using the SC calculated for each image during the time course, subtracted from a duplicated single frame offset image to yield the change in surface distribution with time (dSD/dT) as previously described.^24^ dSD/dT expressed relative to SC of the corresponding, unprocessed frame produces dSD/dT/SC; a measurement of the rate of change of platelet capture ranging from a numerical value of 1 for 100% rolling and 0 for static, adherent platelets.

### 2.8 Immunofluorescence

All samples were imaged using an Olympus UplanFLN 40 × NA1.30 oil immersion objective and a field size of 360 × 360 μm.

#### 2.8.1 VWF adhesion to collagen fibers

Glass slides were coated with 100 µg/mL type I collagen for 1 hour at 24°C. Slides were then washed with PBS and overlaid with 5 µg/mL intact or cleaved VWF for 1 hour at 24°C. After washing, bound VWF was detected using rabbit anti‐VWF (1:2000 dilution; abcam) overnight at 4°C and fluorescein isothiocyanate (FITC)‐conjugated anti‐rabbit (Sigma, UK) for 1 hour at 24°C.

#### 2.8.2 GpIbα and P‐selectin immunofluorescence

Following whole blood perfusion experiments, adherent platelets were fixed with 10% neutral buffered formalin for 30 minutes. The slides were blocked with 1% BSA for 1 hour prior to the addition of 1:1000 dilution anti‐human GpIbα (AF4067; R&D Systems, Abingdon) or anti‐P‐selectin (ab6632; abcam) overnight at 4°C. Slides were washed and anti‐sheep‐FITC (F5137; Sigma) or anti‐mouse Alexa‐647 (115‐606‐008; Jackson ImmunoResearch) conjugated secondary antibodies added for 1 hour at 24°C prior to washing. Images were then exported to ImageJ1.35 for analysis. Overall fluorescence was measured as Mean Gray area. Images were subjected to thresholding in order to obtain fluorescent surface area.

For original data, please contact rwf10@cam.ac.uk.

## 3 RESULTS

**Figure 1:**
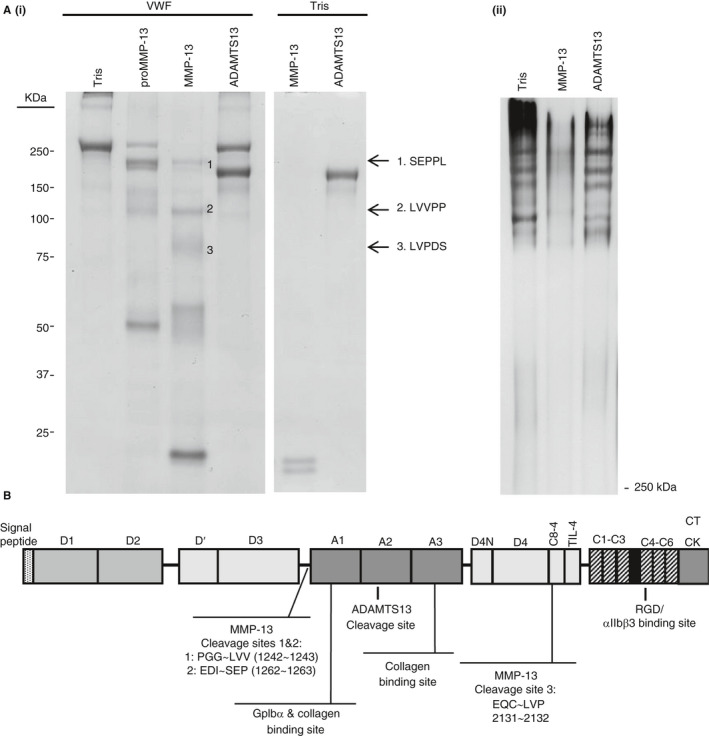
Cleavage of von Willebrand factor (VWF) by matrix metalloproteinase‐13 (MMP‐13). A, SDS‐PAGE of cleaved VWF samples. MMP‐13 but not ADAMTS13 at a concentration of 1.5 µmol/L was able to cleave purified human VWF (0.2 mg/mL) after 2 hours at 37°C. Degradation products were analyzed by (i) 12% reducing SDS‐PAGE and (ii) overnight separation of high molecular weight multimers on 15% acrylamide gels at 4°C. B, Schematic representation of MMP‐13 cleavage sites on VWF. Sequence analysis of MMP‐13 cleavage sites revealed two N‐terminal to the A1 domain and one within the C8‐4 domain. The ADAMTS13 (a disintegrin and metalloprotease with thrombospondin motif member 13) cleavage site within the A2 domain is also marked for reference

**Figure 2:**
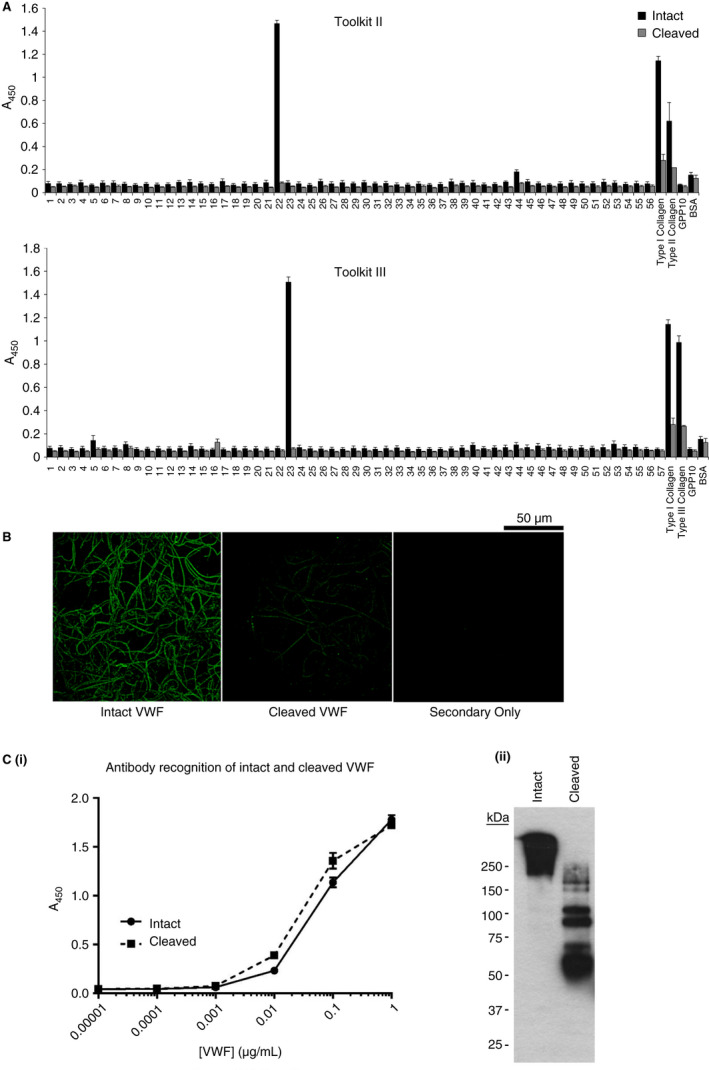
Antibody recognition of von Willebrand factor (VWF) and adhesion of VWF to collagen and collagen Toolkit peptides. A, Plates were coated with 10 µg/mL Toolkit II peptides, fibrillar type I collagen, and the negative binding peptide GPP_10_. Intact VWF (black bars) and cleaved VWF (gray bars) at a concentration of 5 µg/mL were allowed to adhere to the peptides for 1 hour at room temperature. Data represent mean A450 ± SE of three experiments. B, Intact and cleaved VWF were allowed to adhere to fibrillar collagen type I coated glass slides for 1 hour at room temperature and detected using an anti‐VWF primary and FITC‐linked secondary antibody. C, Detection of intact and cleaved VWF via (i) ELISA and (ii) Western blot. Images are representative of three replicate experiments

Incubation with MMP‐13 but not ADAMTS13 resulted in the cleavage of VWF under static conditions (Figure 1A). After incubation for 2 hours at 37°C, ADAMTS13 remained intact whereas MMP‐13 had completely autolyzed. ProMMP‐13 exhibited some proteolytic activity against VWF (due to a degree of autolysis into active MMP), though as expected cleavage was less aggressive than that observed for the active form of the enzyme. MMP‐13 was also able to cleave VWF incubated in human plasma and so is active in vitro (as seen by an increased number of degradation products in Figure S1B in supporting information). Sequence analysis of the main degradation products (bands designated 1, 2, and 3 in Figure 1A) revealed cleavage sites of PGG ~ LVV, EDI ~ SEP, and EQC ~ LVP; the first two of which are located in close together just before the A1 domain at residues 1243 and 1262, respectively. The third is located in the C8‐4 region of the D domain cluster (Figure 1B). Low molecular weight bands (5‐10 kDa) of fully unstable autolyzed (AL)MMP‐13 but not the more stable ADAMTS13 are also visible following incubation. Solid phase binding assays to collagen and Toolkit peptides revealed that as expected, intact VWF was able to bind to collagens I, II, and III and its target Toolkit peptides II‐22 and III‐23;^25^ however, cleavage of VWF by MMP‐13 completely abolished adhesion to both Toolkit peptides and greatly reduced adhesion to all collagen types tested (Figure 2A). Fluorescence microscopy of VWF bound to collagen I fibers corroborated these results, with the binding of MMP‐13‐cleaved VWF greatly reduced over that of its intact counterpart (Figure 2B). Although cleaved VWF contains some (AL)MMP‐13, this form of the enzyme is unable to unwind and therefore to cleave the collagen substrate. In addition, although VWF does adhere to MMP‐13 (and to a lesser extent its composite domains) as seen in Figure S1A, this interaction does not impede the binding of VWF to its target Toolkit peptides (Figure S1B). Antibody affinity assays revealed no difference in detection between intact and MMP‐cleaved VWF (Figure 2C).

**Figure 3:**
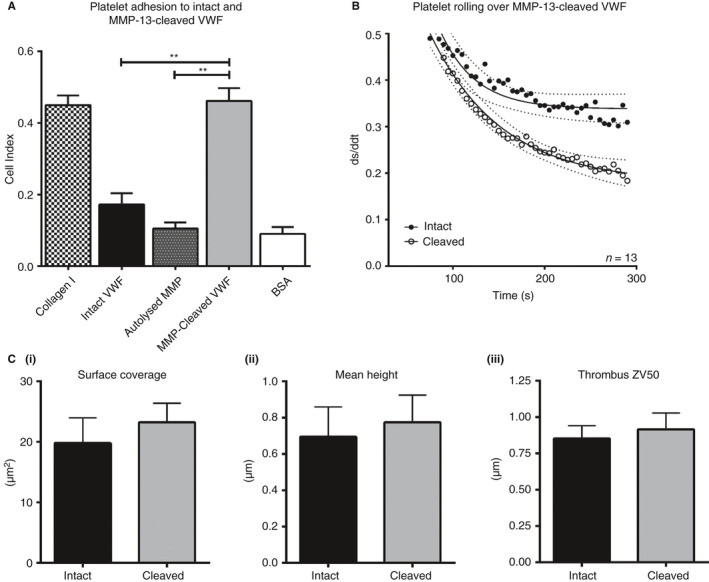
Platelet adhesion, rolling, and thrombus formation on intact and cleaved von Willebrand factor (VWF). A, Binding of washed platelets to intact and cleaved VWF in xCELLigence solid phase binding assays. Collagen type I, autolyzed matrix metalloproteinase 13 (MMP‐13) and bovine serum albumin (BSA) were used as control substrates. ** *P* < .001 (one‐way analysis of variance [ANOVA] and post‐hoc Tukey honestly significant difference [HSD] test). Data represent mean standard error (SE) of 12 separate donors. B, Whole blood platelet rolling at a shear rate of 1000 seconds on intact (black circles) and cleaved (open circles) VWF. ds/ddt is used as a measurement of platelet motility with a measurement of 1 and 0 indicating fully rolling and fully adherent platelets, respectively. Data represent mean ± SE of 13 separate donors. 95% confidence intervals are also shown for the best fit lines, calculated by linear regression (Prism 6). C, Deposition of platelets on MMP‐13 VWF in whole blood under flow conditions. Surfaces were prepared and blood was drawn through the perfusion chamber for 5 minutes as described in Methods. Results shown for surface coverage (i), mean height (ii), and ZV50 (iii) are mean values of 13 separate donors ± SE

**Figure 4:**
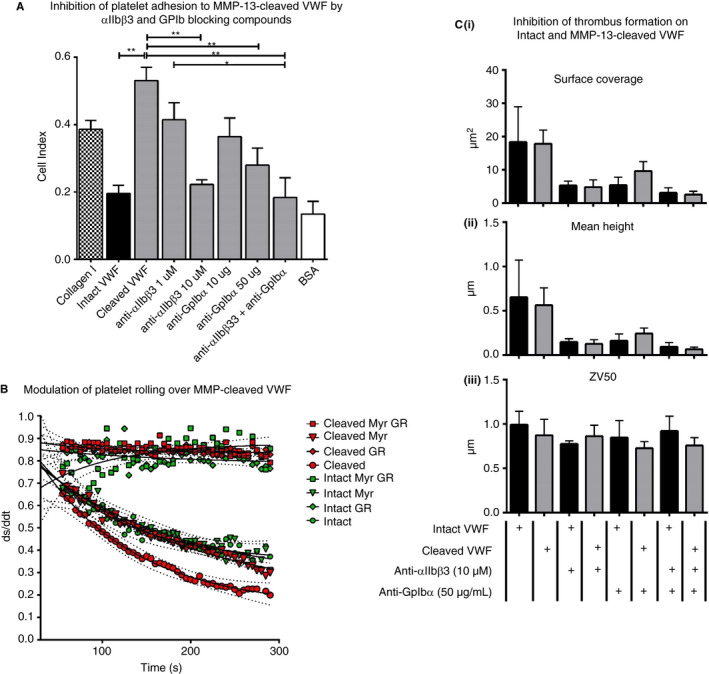
Inhibition of platelet adhesion, rolling, and thrombus formation on intact and cleaved von Willebrand factor (VWF) using anti‐αIIbβ3 and anti‐GpIbα compounds. A, Binding of washed platelets to intact and cleaved VWF in xCELLigence solid phase binding assays. Collagen type I and bovine serum albumin (BSA) were used as control substrates. * *P* < .05, ** *P* < .01 (one‐way analysis of variance [ANOVA] and post‐hoc Tukey honestly significant difference [HSD] test). Where indicated, washed platelets were pre‐incubated with 1 or 10 µmol/L anti‐αIIbβ3 and/or 10 or 50 µg/mL anti‐GpIbα compounds. B, Whole blood platelet rolling at a shear rate of 1000 seconds on intact (green symbols) and cleaved (red symbols) VWF, respectively. Where indicated, washed platelets were pre‐incubated with 10 µmol/L anti‐αIIbβ3 and/or 50 µg/mL anti‐GpIbα compounds. C, Results are shown for (i) surface coverage, (ii) mean height, and (iii) ZV50. Results are mean values of four separate donors ± SE

Washed platelet adhesion to cleaved VWF was significantly higher than that observed for intact VWF or autolyzed MMP‐13 alone, which supported only low levels of binding (Figure 3A; *P* ˂ .01; one‐way analysis of variance [ANOVA] with post‐hoc Tukey HSD test). Whole blood platelet rolling on cleaved VWF was also significantly slower than on intact VWF (Figure 3B) although interestingly, platelet thrombus surface coverage, height, and ZV50 were not differentially affected (Figure 3Ci‐iii). In separate stand‐alone experiments, washed platelet adhesion, rolling, and thrombus deposition on intact VWF compared with intact VWF co‐coated with autolyzed (AL)MMP‐13 were not significantly different (Figure S2A‐Ci‐iii [respectively] in supporting information). Blockade of platelet receptors αIIbβ3 and GpIbα with 10 µmol/L GR 144053 and 50 µg/mL Myr‐Ser‐Ile‐Arg‐Tyr‐Ser‐Gly‐His‐Ser(PO_3_H_2_)‐Leu (myr‐SIRYSGHS(P)L), respectively significantly reduced platelet adhesion to cleaved VWF (Figure 4A) with both inhibitors combined exerting maximal effect, returning adhesion to the intact VWF baseline (*P* < .01; one‐way ANOVA with post‐hoc Tukey HSD test). In platelet rolling experiments, pre‐incubation of whole blood with 50 µmol/L (myr‐SIRYSGHS(P)L) caused a reversion to the intact VWF phenotype (Figure 4B), indicating that the increase in adhesion and slower rolling rates are due primarily to a stronger/longer GpIbα:platelet attachment. Platelet surface coverage, thrombus height, and ZV50 were not significantly altered between parallel assays on intact and cleaved VWF.

**Figure 5:**
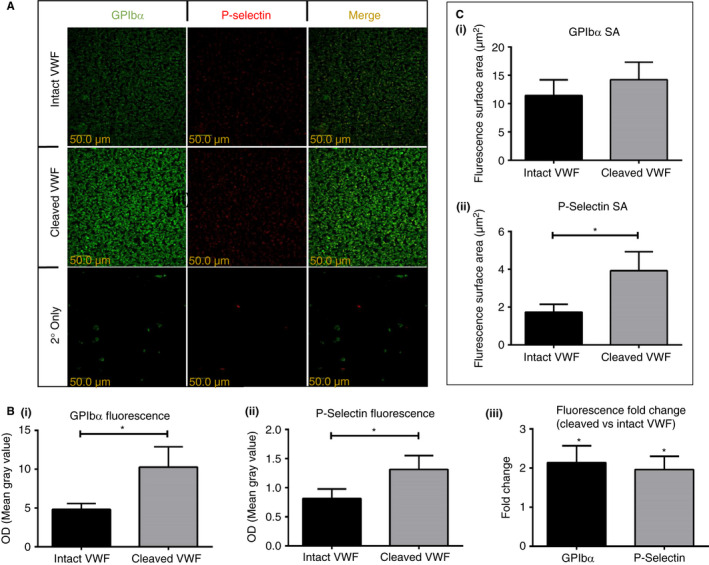
GpIbα and P‐selectin expression on platelets following whole blood flow experiments. Whole blood was drawn through a flow chamber for 5 minutes over intact or cleaved von Willebrand factor (VWF) using a syringe pump to generate a wall shear rate of 1000 seconds^-1^, corresponding to arteriolar conditions. Adherent platelets were then fixed in formalin prior to GpIbα and P‐selectin detection using FITC Alexa‐647 conjugated secondary antibodies respectively. A, Representative images of protein expression. B, Overall fluorescence and fold change of GpIbα and P‐selectin expression were calculated using Mean Gray value in ImageJ1.35. C, Fluorescent surface area of GpIbα and P‐selectin expression were determined following thresholding of images in ImageJ1.35. Data are the mean ± SE of nine separate donors. **P < .005 (two‐tailed paired *t*‐test)

Immunofluorescence experiments post blood flow revealed that both platelet GpIbα and P‐selectin expression were significantly increased (Figure 5A) on platelets adherent on cleaved versus intact VWF (each *P* < .05, two‐tailed paired *t*‐test; Figure 5Bi and ii). Both GpIbα and P‐selectin were upregulated approximately two‐fold in response to adhesion to cleaved VWF (Figure 5Biii). As expected, the GpIbα fluorescent surface area on cleaved and intact VWF were very similar (Figure 5Ci), indicating the same number of adherent platelets expressing the receptor, albeit by differing amounts (and thus fluorescence intensity) between conditions (Figure 5Bi). A significant increase (*P* = .02) in fluorescent surface area corresponding to an increase in the number of platelets expressing P‐selectin was observed, however (Figure 5Cii), indicating a greater degree of platelet activation and reinforcing our solid‐phase binding assay observations.

**Figure 6:**
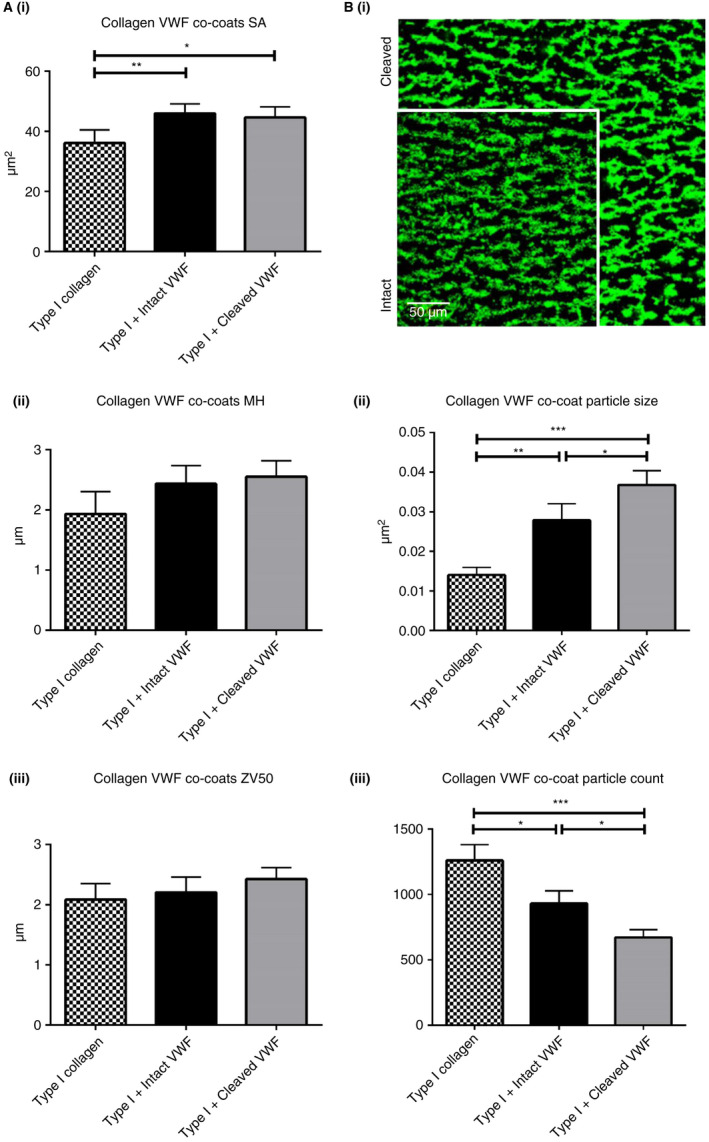
Platelet adhesion and thrombus deposition on von Willebrand factor (VWF) co‐coated with fibrillar type I collagen. A, Surface coverage (i), mean height (ii), and ZV50 (iii) of thrombi formed on 10 µg/mL intact and cleaved VWF co‐coated with 100 µg/mL type I collagen fibres. Surface coverage (i), mean height (ii), and (iii) ZV50 are the mean ± standard error (SE) of nine different donors;*P < .05 (one‐way analysis of variance [ANOVA] and Tukey post‐hoc honestly significant difference [HSD] test). Representative images of thrombi formed on intact and cleaved VWF co‐coated with type I collagen fibers as described above. Images are of identical scale with one cut away to reveal the other. B, Mean particle count and size were obtained from ImageJ1.35‐thresholded images. *P < .05; **P < .01; ***P < .001 (two‐tailed paired *t*‐test). Data are the mean ± SE of nine different donors

**Figure 7:**
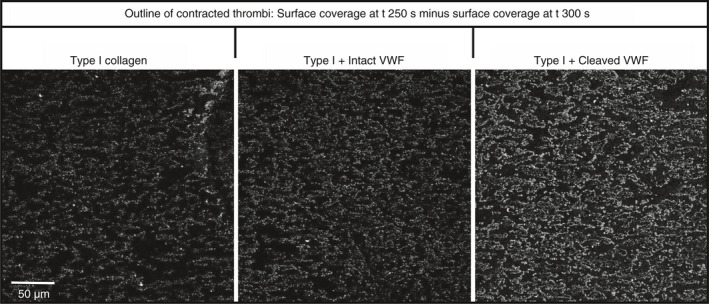
Representative images of thrombus contraction on intact and cleaved von Willebrand factor (VWF) co‐coated with fibrillar type I collagen. Whole blood at a shear rate of 1000 seconds^-1^ was drawn over intact and cleaved VWF co‐coated with fibrillar type I collagen for 5 minutes with an image of field size 360 × 360 μm taken once every 5 seconds. Following acquisition, images were thresholded in ImageJ1.35 and the final image, at t = 300 seconds, was subtracted from that obtained at t = 250 seconds to reveal an outline corresponding to contracted thrombus

Co‐coating collagen I fibers with intact and cleaved VWF prior to whole blood flow resulted in a slight but significant increase in surface coverage relative to collagen I alone (intact VWF, *P* = .0083 and cleaved VWF, *P* = .0143; Figure 6Ai). No significant change was observed in mean thrombus height or ZV50 (Figure 6Ai and ii). Thrombus morphologies, however, differed considerably between collagen co‐coated with intact and cleaved VWF (Figure 6Bi). Image analysis revealed that co‐coating VWF with collagen produced thrombi with significantly larger particle size (Figure 6Bii) and correspondingly lower particle count than with collagen alone (Figure 6Biii), but co‐coating with cleaved VWF led to a further increase in particle size, significantly greater than collagen I, both alone and co‐coated with intact VWF (Figure 6Bii and iii). In essence, thrombi formed on co‐coated cleaved VWF were less fragmented, and although covering the same surface area as those on co‐coated intact VWF, formed larger, more contiguous, denser thrombi. We theorized that such apparently tighter aggregates might occur due to greater platelet contraction within the thrombi. When the final binary image, obtained at t = 300 seconds, was subtracted from that at t = 250 seconds, an outline corresponding to the degree of contraction of thrombi was clearly visible (Figure 7). This effect was most marked for co‐coatings of collagen I with cleaved VWF, then with intact VWF, which in turn was greater than collagen I alone, indicating that the highest degree of retraction occurred with exposure to cleaved VWF.

## 4 DISCUSSION

Platelets adhere to sites of vascular injury to halt bleeding. Membrane receptors that promote adhesion are essential for platelet tethering and arrest on the exposed endothelial surface. The initial rolling of platelets on VWF under shear is mediated largely by its interactions with the glycoprotein‐Ibα (GPIbα) subunit of the platelet GPIb‐IX‐V complex and subsequently with the integrin αIIbβ3. αIIbβ3, the primary platelet fibrinogen receptor, can also bind the VWF C4‐8 module,^26^ while glycoprotein GpIbα binds to the A1 domain of VWF, both attached to platelets and immobilized on collagen exposed in the vessel wall.^27^

MMP‐13 is a collagenolytic protease whose presence and activity is upregulated in unstable atherosclerotic lesions, and which has a predominant role in mediating plaque rupture.^6^ Most MMPs, however, have many substrates and here we show that MMP‐13 can also cleave VWF under static conditions. This ability sets it apart from ADAMTS13, a circulating metalloproteinase which cleaves highly thrombotic ultra large VWF multimers into smaller fragments but only when the A2 domain of VWF is accessible under shear conditions.^28^ ADAMTS13 cleavage of unfolded VWF safeguards against the accumulation of ultra large VWF multimers and the subsequent formation of platelet aggregates that may occlude arterioles and capillaries.^29^ Here we show that MMP‐13 cleaves VWF at three main sites, two just prior to the A1 domain, and the third in the C8‐4 domain. Under inflammatory conditions and following plaque rupture, this multisite cleavage of VWF is likely to result in the presence of VWF in various stages of proteolysis and has distinct consequences for collagen and platelet binding. The ability of such cleaved VWF to bind to collagen and to Toolkit peptides was virtually abolished, while its platelet binding capacity increased. VWF binds to collagen primarily through its A3 domain, although in its absence, the A1 domain can substitute for A3 under flow conditions.^30^ Proteolytic degradation of VWF has previously been shown to impair its binding to microtiter plates coated with human collagen,^31^ and VWF multimer defects, depending upon the degree of structural abnormality, often result in low collagen binding.^32^ It would therefore appear that cleavage of VWF, for example by MMP‐13, can result in altered protein organization, which masks the collagen binding motifs in the A1 and A3 domains.

In contrast, MMP‐13‐cleaved VWF was able to support a much higher degree of platelet adhesion under static conditions than intact VWF. Cleaved VWF also supported slower platelet rolling than intact VWF, indicating that cleaved VWF adopts a conformation that supports a longer or stronger interaction with platelets. The A1 domain of VWF forms an autoinhibitory module that masks the A1 domain.^33^ Cleavage of VWF just prior to the A1 domain may release it from protection by the D3 domain and/or destabilize the N‐terminus of the A1 domain to expose the GPIbα binding site.^34^ In addition, the sequence length of the N‐terminal flanking region preceding the A1 domain has been shown to affect the ability of the A1 domain to interact with platelet GpIbα.^35^ Cleavage of VWF by MMP‐13 in this region could therefore be expected to modulate this interaction. Interestingly, limited proteolysis of VWF by trypsin also yields a conformational change in VWF that promotes ristocetin‐induced GpIbα binding, which may occur via a similar mechanism.^36^ The actions of VWF are mediated via two main platelet receptors, GpIbα (as part of the GPIb‐V‐IX complex) and αIIbβ3; with the former mediating platelet adhesion and the latter orchestrating the resulting platelet aggregation. Upon interaction with VWF, GpIb‐V‐IX initiates transmembrane signalling events, which result in αIIbβ3 activation and platelet aggregation.^27^ Platelets adherent to coated “solid phase” VWF express a concentration‐dependent increase in GpIbα expression, but not αIIbβ3.^37^ Here we show that cleavage of VWF augments this effect, increasing GpIbα expression levels on adherent platelets over those observed with intact VWF. Tethering of platelets under high shear almost exclusively via GpIbα makes this receptor pivotal to hemostasis.^27^ The slower rate of whole blood platelet rolling on cleaved VWF was nullified following GpIbα inhibition, and, after rolling experiments, platelets adherent to cleaved VWF displayed correspondingly (two‐fold) higher levels of both GpIbα and P‐selectin expression, suggesting a greater degree of GpIbα‐mediated platelet activation. These observations were supported by our solid phase binding assays in which antagonists of both GpIbα and αIIbβ3 reduced washed platelet binding to intact VWF levels.

Although platelets roll on immobilized VWF, they require ligands in exposed connective tissue for firm adhesion and aggregation. VWF presented on the vessel wall is usually co‐localized with newly exposed collagen.^38^ Although all of the platelets within a thrombus are likely to interact with VWF, only those closely packed in the center normally become pro‐coagulant and P‐selectin positive.^39^ Collagen and VWF are known to act synergistically in supporting platelet adhesion at the site of injury.^14^ Although MMP‐13‐cleavage of VWF abolishes its ability to bind to fibrillar collagen, cleaved VWF is still able to adhere to platelet GpIbα and αIIbβ3 and would co‐localize with the exposed collagen. VWF also interacts with laminin, fibronectin, thrombospondin, and vitronectin within the extracellular matrix to maintain a substrate platform during thrombus formation. The question was therefore whether slowing of platelet rolling and a greater degree of platelet activation in the presence of cleaved VWF is sufficient to result in greater/firmer platelet adhesion and the formation of larger thrombi. Co‐coating intact and cleaved VWF with fibrillar collagen I resulted in a small but significant increase in thrombus surface area; however, the most striking observation was the change in thrombus morphology between conditions. Platelet aggregates formed on cleaved co‐coated VWF were significantly larger than those on co‐coated intact VWF and collagen alone, with far fewer separate platelets or smaller outlying thrombi. These larger, more amalgamated thrombi translated into an overall increased mean particle size and correspondingly lower particle count. GpIb‐V‐IX and αIIbβ3 are known to have a large role in platelet mechanobiology; the adhesion of VWF to GpIbα can trigger mechanotransduction and platelet activation by enhancing the drag force applied on the cell‐surface receptor^40^ and both receptors work together to mediate platelet shape change and contraction during activation and aggregation.^41^ We hypothesized that these more dense thrombi, likely to contain a larger proportion of active platelets, may result in a greater degree of clot contraction in the latter stages of thrombus formation. Image subtraction of end‐stage thrombi from those obtained 50 seconds earlier confirmed that the platelet aggregates on cleaved co‐coated VWF contracted more than those on intact co‐coated VWF or collagen I alone. Platelet‐driven clot contraction is crucial for hemostasis, wound healing, and the restoration of blood flow past otherwise obstructive thrombi.^42^ Contraction, however, can also confer a resistance to clot lysis therapies.^43^ Atherosclerotic plaque rupture, thrombosis, and its associated pathologies—including stroke, reperfusion injury, and hemorrhagic transformation—are associated with an upregulation of MMP activity, with MMP‐9 and ‐13 implicated in the early pathology of stroke progression.^9^, ^10^, ^44^ Here we demonstrate that the cleavage of VWF by MMP‐13 perturbs two distinct processes integral to the process of thrombus formation; on the one hand inhibiting VWF adhesion to collagen, but increasing platelet activation in thrombi formed in flowing whole blood. It may be that in this way MMP‐13 plays its role in the pathology of ischemic stroke; mediating the formation of highly contractile thrombi which may be more resistant to lysis therapies and which are also more prone to detachment from the collagen‐rich vessel wall. MMP‐13 would appear therefore to modulate the architecture of thrombi around the site of plaque rupture to increase risk of stroke.

## CONFLICTS OF INTEREST

We declare that Joanna‐Marie Howes, Vera Knäuper, Jean‐Daniel Malcor, and Richard W. Farndale have no conflicts of interest to disclose.

## AUTHOR CONTRIBUTIONS

Vera Knäuper and Jean‐Daniel Malcor provided essential materials, Richard Farndale designed the research and helped write the manuscript, and Joanna‐Marie Howes designed and performed the research and wrote the manuscript.
