# Supplementary material for: Cleavage by MMP‐13 renders VWF unable to bind to collagen but increases its platelet reactivity
Source: J Thromb Haemost. 2020 Feb 24;18(4):942–54. doi: 10.1111/jth.14729 (PMC8614119; doi:10.1111/jth.14729)
Supplement: Supplementary file 1 [file JTH-18-942-s002.docx]

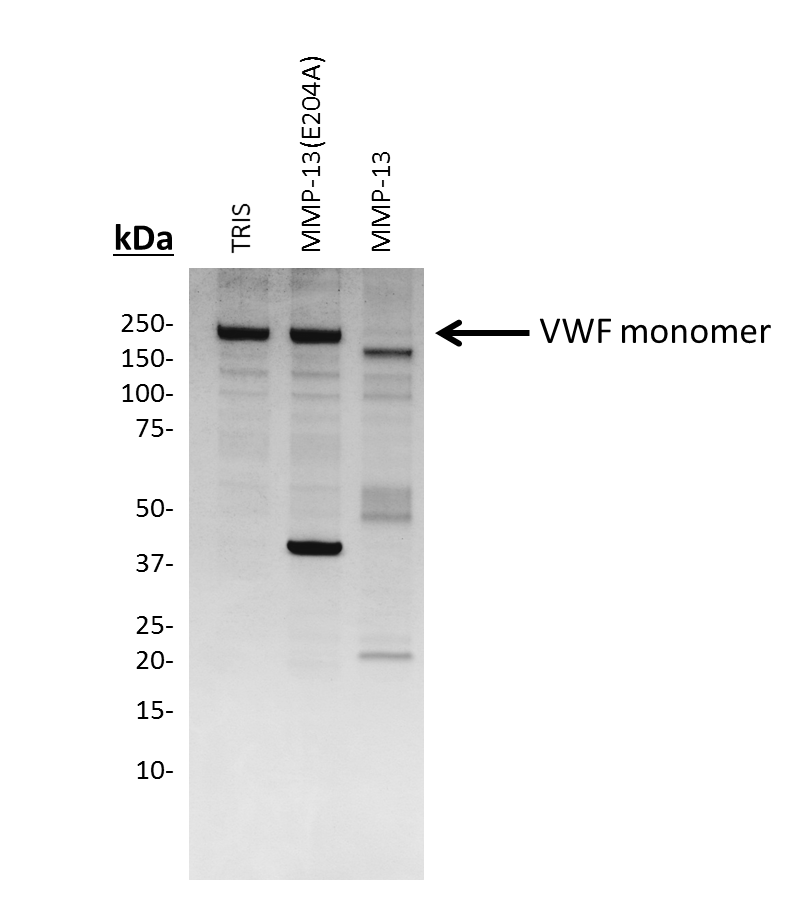

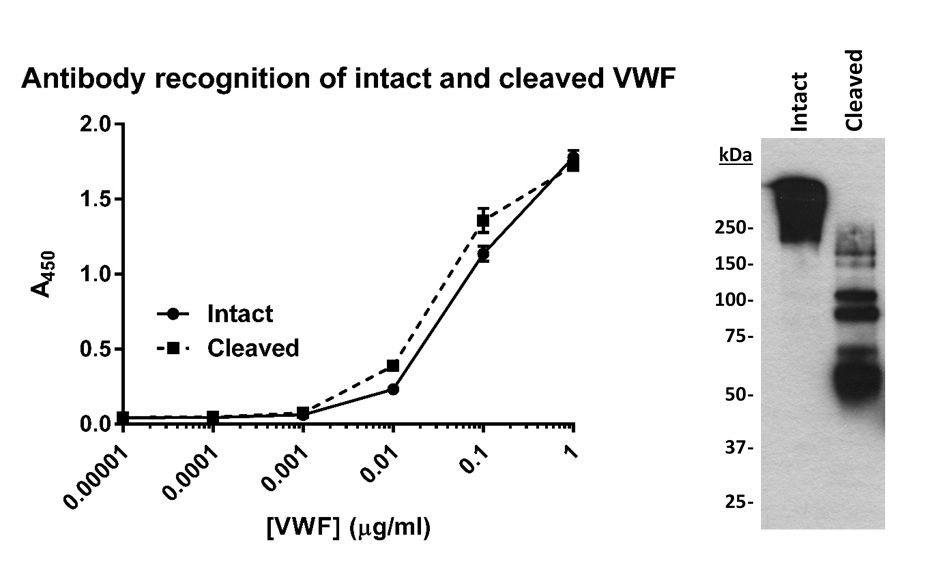


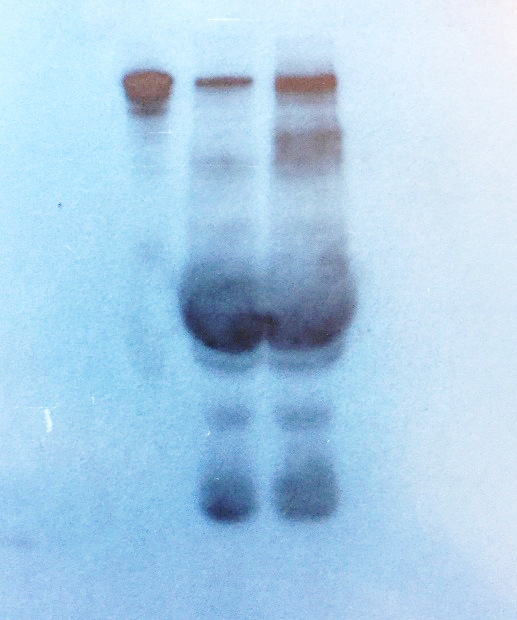


VWF + Tris

Plasma

Plasma + MMP-13

VWF Monomer

A B

C(i) (ii)

VWF Monomer

**Supplemental Figure 1. (A)** Cleavage of VWF by MMP-13 and MMP-13(E204A). SDS-PAGE of cleaved VWF samples. MMP-13 but not MMP-13(E204A) 8 µM was able to cleave purified human VWF (0.2 mg/ml) after 2 h at 37 °C. VWF was also incubated with Tris as a negative control. (B) Cleavage of VWF by MMP-13 in plasma as shown by Western Blot using an anti-VWF antibody. VWF and plasma were incubated with Tris as a negative control. (C) Antibody recognition of intact and cleaved VWF by ELISA and Western blot. (i) Intact (circles) and cleaved (squares) VWF coated on HB 96 well plates was detected with a 1:2000 dilution of anti-VWF antibody raised against the whole molecule. (ii) 500 ng intact or cleaved VWF was subjected to electrophoresis under reducing conditions prior to detection as described in Materials and Methods
