# Supplementary material for: Cleavage by MMP‐13 renders VWF unable to bind to collagen but increases its platelet reactivity
Source: J Thromb Haemost. 2020 Feb 24;18(4):942–54. doi: 10.1111/jth.14729 (PMC8614119; doi:10.1111/jth.14729)
Supplement: Supplementary file 2 [file JTH-18-942-s001.docx]

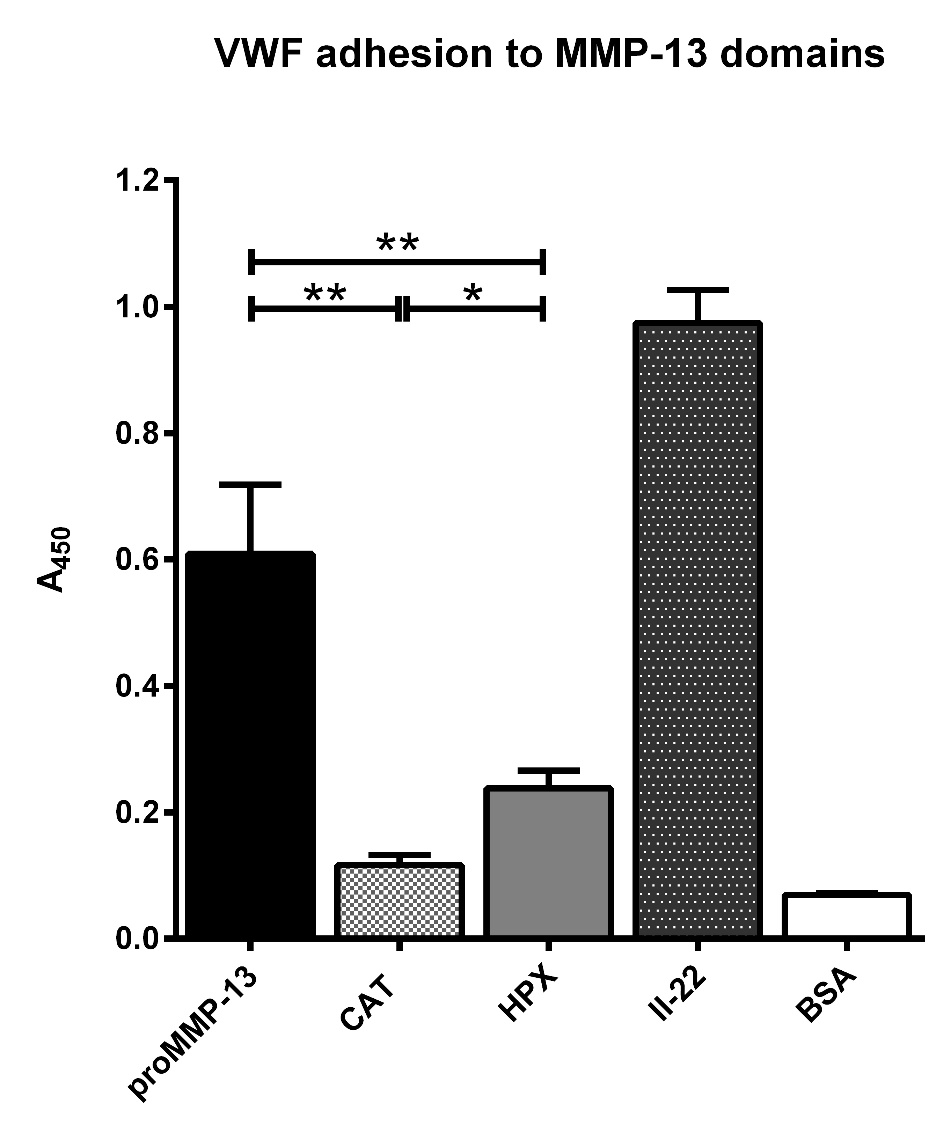

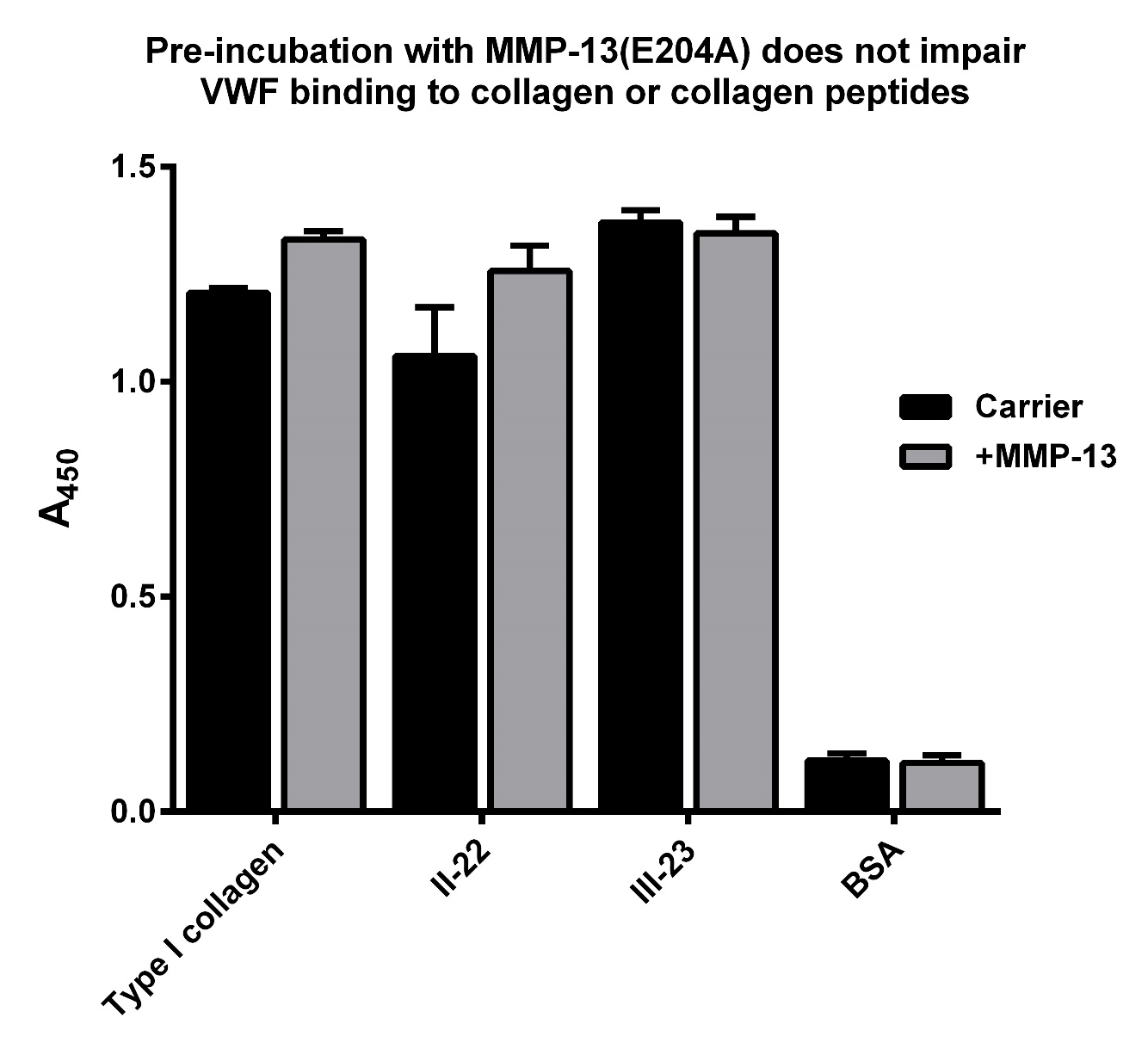


**Supplemental Figure 2. VWF adhesion to MMP-13 domains and binding of VWF to target peptides following pre-incubation with MMP-13.** (A) VWF at a concentration of 5 µg/ml was allowed to adhere to MMP-13 and MMP-13 domains for 1 h at room temperature. Data represent mean A450 ± S.E. of three experiments. BSA was used as a negative control. * p < .05, ** p < .01; (one-way ANOVA and post-hoc Tukey HSD test). (B) VWF (0.2 mg/ml; black bars) or VWF pre-incubated with 83nM MMP-13 for 1h prior (grey bars) were allowed to adhere to collagen type I and target collagen Toolkit peptides. BSA was used as a negative control. Data represent mean A450 ± S.E. of three experiments.

**A B**
