# Supplementary material for: Cleavage by MMP‐13 renders VWF unable to bind to collagen but increases its platelet reactivity
Source: J Thromb Haemost. 2020 Feb 24;18(4):942–54. doi: 10.1111/jth.14729 (PMC8614119; doi:10.1111/jth.14729)
Supplement: Supplementary file 3 [file JTH-18-942-s003.docx]

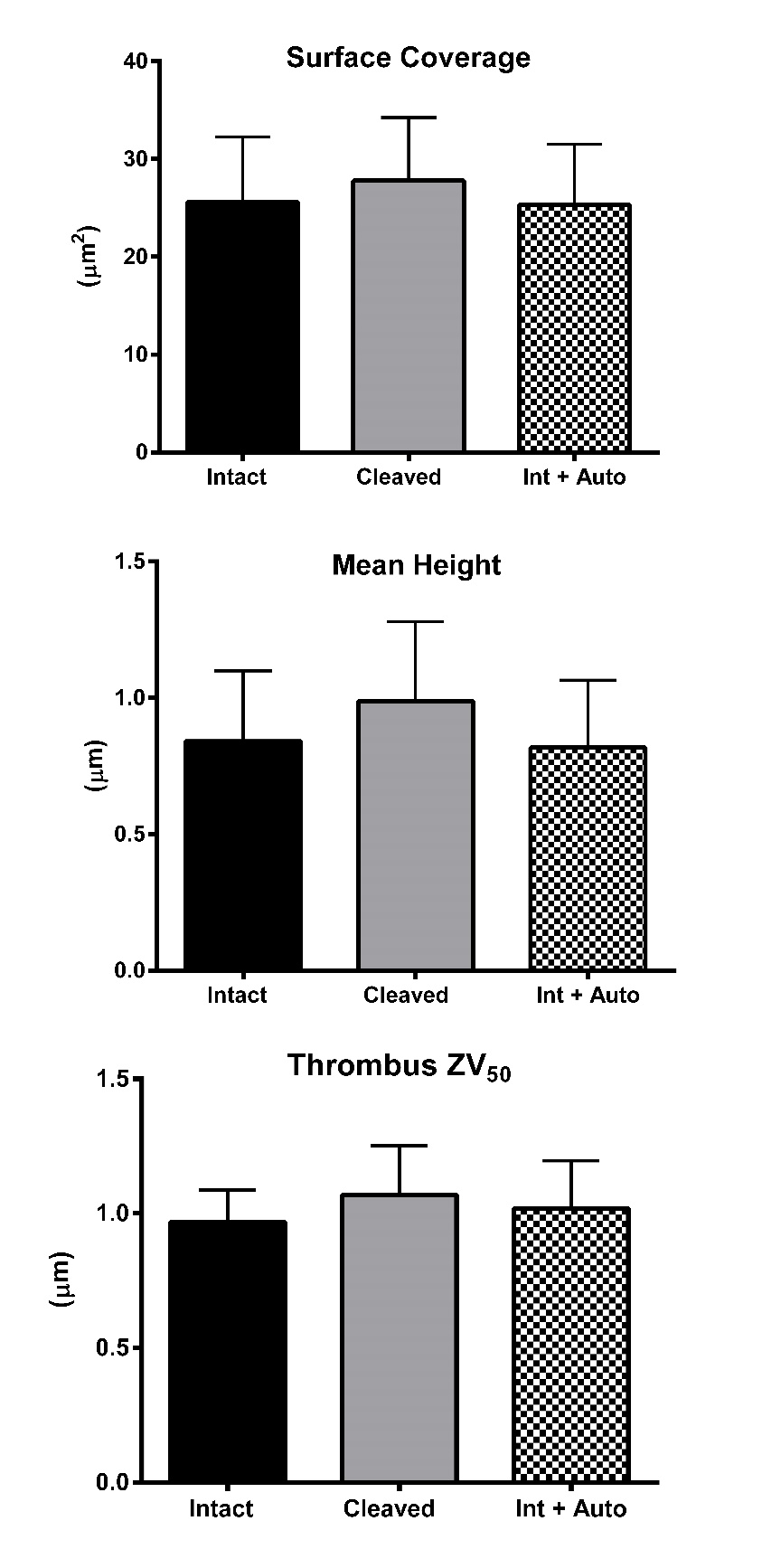

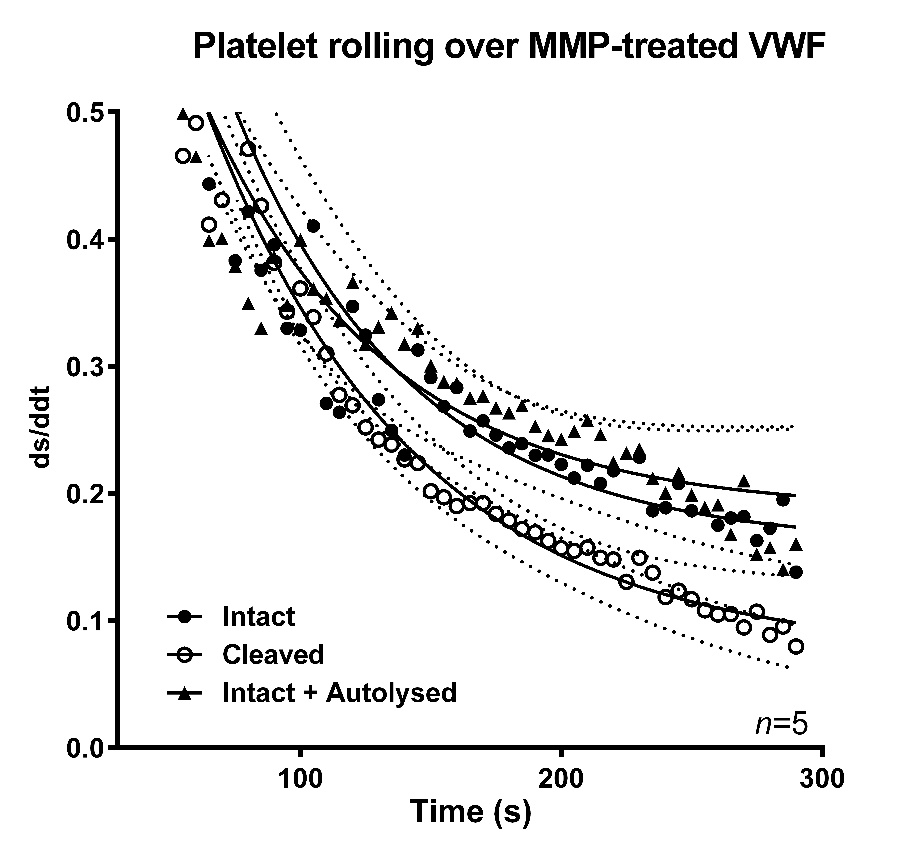

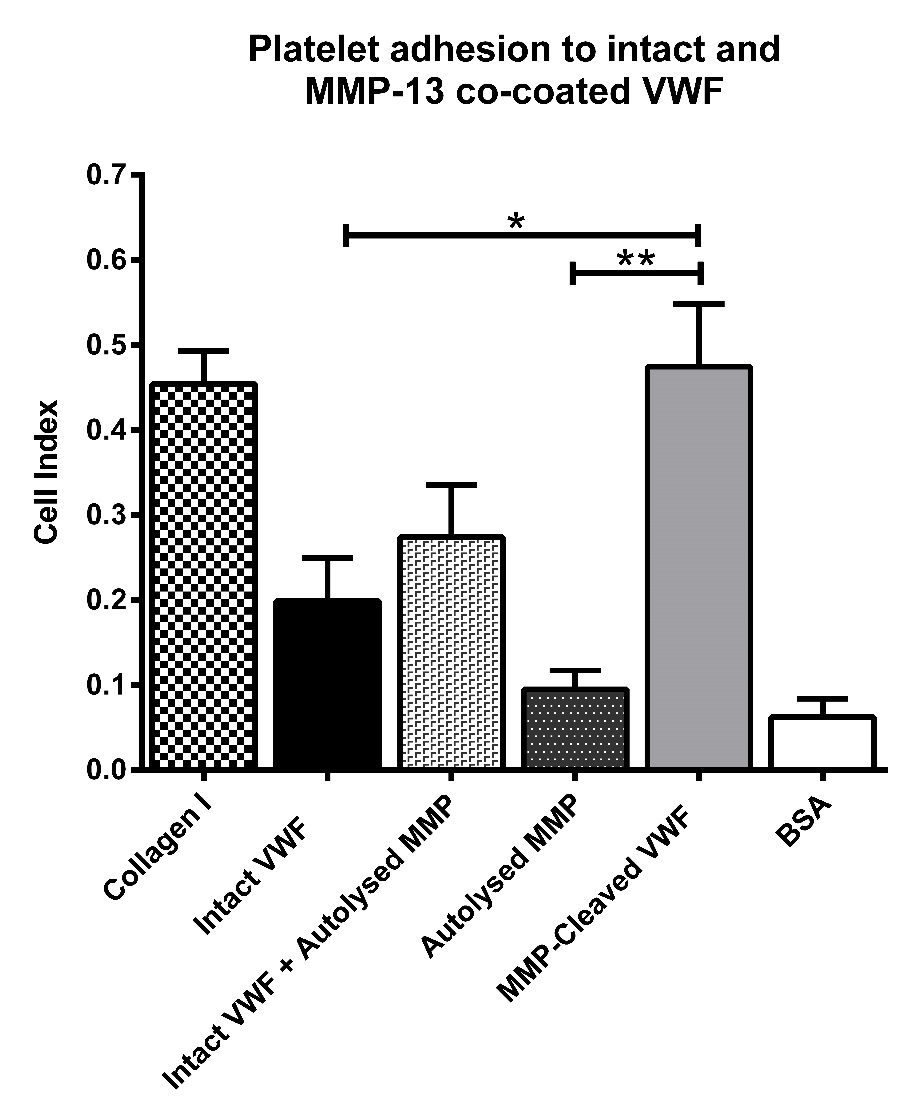


**Supplemental Figure 3. Platelet adhesion, rolling and thrombus formation on cleaved VWF and intact VWF co-coated with autolysed MMP-13.** (A) Binding of washed platelets to intact and cleaved VWF in xCELLigence solid phase binding assays. Collagen type I, autolysed MMP-13, intact VWF co-coated with autolysed MMP-13 and BSA were used as control substrates. * p < .05, ** p < .01; (one-way ANOVA and post-hoc Tukey HSD test). (B) Whole blood platelet rolling at a shear rate of 1000^s-1^ on intact VWF (black circles), intact VWF co-coated with autolysed MMP-13 (black triangles) and cleaved VWF (open circles). (C) Results are shown for (i) surface coverage (ii) mean height and (iii) ZV_50_. Results are mean values of five separate donors ± SEM.

**A C(i)**

**(ii)**

**B**

**(iii)**
